# Mitral valve infective endocarditis in a dialysis patient with a tunneled dialysis catheter and prior MitraClip® implantation: an autopsy case

**DOI:** 10.1186/s12872-023-03176-0

**Published:** 2023-03-24

**Authors:** Takahiro Doi, Atunari Utusgi, Koki Kikuchi, Yoshio Kazuno, Satoshi Yuda

**Affiliations:** 1 Department of Cardiology, Teine Kijinkai Hospital, Sapporo, Hokkaido Japan; 2 Department of Infectious Diseases, Teine Kijinkai Hospital, Sapporo, Hokkaido Japan

**Keywords:** Infective endocarditis, MitraClip®, Elastica-Masson staining

## Abstract

**Background:**

The number of patients with heart disease who can benefit from treatment is continuing to increase due to the widespread use of cardiac implantable devices. Accordingly, the number of cardiac device-related infective endocarditis (CDRIE) cases has been increasing year by year. We report a very rare experience of performing an autopsy on a patient who died of CDRIE at the site of MitraClip ® implantation, which has recently been developed as a treatment option for severe mitral regurgitation. In addition to hematoxylin–eosin (H-E) staining, Elastica-Masson staining in the present case revealed destruction of all of the atrial, trabecular, fiber and myocardial layers.

**Case presentation:**

The patient was hemodialyzed with a dialysis catheter. Hemodialysis treatment was difficult due to functional mitral regurgitation caused by cardiac dysfunction, and the MitraClip® procedure was performed. However, he subsequently developed a fever and dialyzation became difficult again, and he was admitted to the cardiology department.

Echocardiography revealed a large vegetation at the site of MitraClip® implantation and a diagnosis of CDRIE was made. Guidelines recommend removal of the device and surgical intervention. However, considering the patient's general condition, a decision was made at a heart team conference to give priority to antibiotic therapy. However, the patient did not respond to antibiotic therapy and died of septic shock.

**Conclusion:**

To our knowledge, this is the first reported case of CDRIE and death after MitraClip® implantation that resulted in an autopsy. Furthermore, not only H-E staining but also Elastica-Masson staining was performed, and it was confirmed that there was significant valve tissue destruction. In the future, the MitraClip® procedure, even though it is minimally invasive, should be carefully considered in immunocompromised patients.

## Background

Cardiac device-related infective endocarditis (CDRIE) is an infection that has spread to the endocardium, including device leads and heart valves [1, 2]. The main routes of device infection are device pocket infection during implantation surgery and exposure of the lead or catheter to skin surfaces. CDRIE occurs when inflammation spreads from a device infection to the intracardiac space via an intravascular lead.

Risk factors for CDRIE include renal failure (including hemodialysis patients), steroid use, congestive heart failure, hematoma formation in the device pocket, diabetes mellitus, and anticoagulant use [3–5]. *Staphylococcus*, especially coagulase-negative *Staphylococcus* (CNS), is the most common causative organism, accounting for more than half of all CDRIE cases [6, 7]. Device infections may be mixed infections caused by multiple causative organisms, and care must be taken to identify the causative organisms [7, 8]. As with other cases of infective endocarditis (IE), blood cultures and cardiac echocardiography are central to the diagnosis for CDRIE.

The principle of treatment for CDRIE, with reference to the ESC guidelines 2015, is continuous administration of antimicrobial agents and complete removal of the device including the lead [9, 10]. In patients with CDRIE, the rate of IE recurrence is high and prognosis is poor when medical treatment alone is performed without device removal.

In recent years, transcatheter aortic valve implantation** (**TAVI) has been performed in clinical practice as a treatment for severe aortic stenosis in very old patients and those at high surgical risk [11]. However, in patients who underwent TAVI, male gender, diabetes, and moderate to severe residual aortic regurgitation were showed to be significantly associated with an increased risk of IE, and it was also reported that patients who developed endocarditis had higher in-hospital and 2-year mortality rates [12].

Percutaneous catheter mitral valve repair, which is minimally invasive like TAVI, has also been developed as a treatment for patients with inoperable or high-risk mitral regurgitation (MR). MitraClip® (Abbott Vascular, Santa Clara, CA, USA) therapy is currently the most popular therapy. MitraClip® therapy is very promising for secondary MR with heart failure [13–15].

There have been limited reports of CDRIE in patients treated with MitraClip® [16, 17] and even fewer reports of autopsy cases. In addition, there have been few case studies in which Elastica-Masson staining was performed to assess the degree of valve destruction caused by infective endocarditis.

In this article, we report a very rare experience of treating a case of IE at the implantation site of MitraClip® and performing an autopsy on a patient who unfortunately died.

## Case presentation

The patient, a 66-year-old male, had been undergoing outpatient hemodialysis treatment for one year for end-stage renal failure with autosomal dominant polycystic kidney disease. Transthoracic echocardiography performed at the start of hemodialysis showed a left ventricular ejection fraction of approximately 30% and severe MR from between the middle scallop of anterior leaflet (A2) and posterior leaflet (P2). The patient had functional MR due to valve ring enlargement and tethering. Tricuspid regurgitation was mild. There was no vegetation on the mitral valve or other valves. Hence, Bio-Flex® Tesio® Cath for long-term hemodialysis was inserted through the left subclavian vein and the patient was hemodialyzed using it as a blood access.

The patient initially consulted the department of cardiology because of decreased left ventricular contraction shown by transthoracic echocardiography, shortness of breath, cardiac enlargement and pulmonary congestion on a chest X-ray. Although coronary angiography showed that there was no significant stenosis, transthoracic echocardiography revealed a shallow valve coaptation and marked progression of tethering MR due to left ventricular dilation. The tricuspid valve coaptation was also very shallow and tricuspid regurgitation was severe. According to the 2020 guidelines of the Japanese Circulation Society, the patient had secondary MR, which was very frail and difficult to operate on. Transesophageal ultrasonography showed that the mitral valve morphology was suitable for the MitraClip® procedure, and intervention was expected to relieve the symptoms. In the heart team conference with a cardiovascular surgeon, intervention with MitraClip® was recommended, and percutaneous catheter mitral valve repair was performed. A 1 Clip (MITRACLIP® NT Device) was implanted between the middle scallop of the anterior leaflet (A2) and the posterior leaflet (P2) of the mitral valve after preoperative administration of antibiotics to prevent surgical site infection. There were no intraoperative complications, including adverse events that impaired valve function. MR improved from severe to mild after mitral valve junction repair with placement of a single MitraClip® between the middle scallop of the anterior leaflet and the middle scallop of the posterior leaflet.

**Fig. 1:**
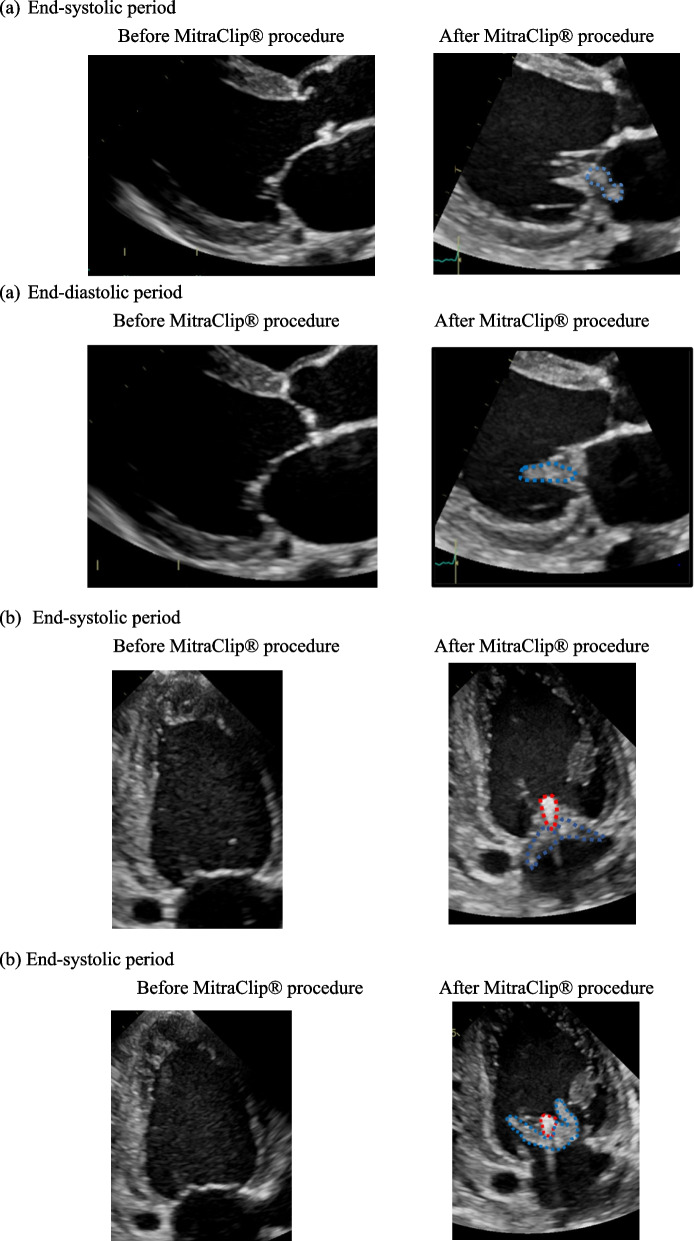
Giant vegetation that had adhered to the MitraClip® was observed when compared to before the MitraClip® procedure. The vegetation had extended to the medial site and lateral site of the native mitral valve and was mobile. The giant vegetation moved into the left ventricular cavity during the diastole period and into the left atrial cavity during the systole period. **a** Parasternal view. Blue dotted line: vegetation. **b** Two-chamber view. Red dotted line: MitraClip®. Blue dotted line: vegetation

The patient continued to have good fluid management on outpatient hemodialysis at another hospital after discharge from our hospital. However, 8 months after MitraClip® implantation, the patient had a fever of 38 °C or higher and hypotension, and dialysis could not be performed even with the use of continuous intravenous administration of noradrenaline during dialysis. He was referred to our hospital with suspicion of septic shock. MRSA was cultured from all blood cultures and also from the tip of the Bio-Flex® Tesio® Cath, which was used as a hemodialysis blood access. Transthoracic echocardiography performed in our hospital showed a large vegetation at the site of the MitraClip® implantation. The vegetation had extended to the medial site and lateral site and was mobile. MR was mild to moderate with outflow from both sides of the clip. (Fig. 1). Head magnetic resonance imaging performed as a screening test also showed multiple cerebral infarctions. After admission, blood culture tests were performed, and methicillin‐resistant *Staphylococcus aureus* (MRSA) was cultured in all three sets. The diagnosis was CDRIE at the site of MitraClip® implantation, and the causative organism was MRSA based on the clinical course and examination.

During the course of the disease, the patient developed disturbance of consciousness, which was suspected to be another cerebral infarction caused by embolization from the vegetation. Transthoracic echocardiography showed that the left ventricular ejection fraction had decreased to about 10%, and adequate water removal with dialysis treatment was difficult. A heart team conference was held with the cardiovascular surgeon, and it was decided to perform only conservative treatment with antibiotics, gentamicin and vancomycin, and not to perform surgery.

**Fig. 2:**
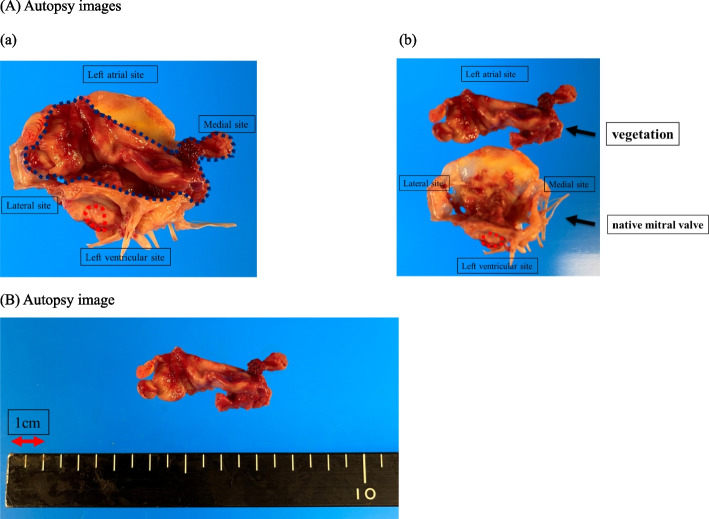
The autopsy findings were similar to the findings of transthoracic echocardiography, showing a large vegetation on the left atrial side. **A** Dorsal view of the vegetation and mitral valve. **a** Image of the giant vegetation attached to the mitral valve. **b** Image of the mitral valve and the giant vegetation isolated from the mitral valve. **B** The size of the giant vegetation was measured to be about 5 cm in width and 1.5 cm in length. Red dotted line: MitraClip®. Grey dotted line: vegetation

On the 7th day, the patient died of peripheral circulatory failure due to septic shock. After his death, we received consent from his family to perform an autopsy. The autopsy specimen showed a large vegetation of 5 cm in size. The vegetation had spread to the left and right sides around the tip of the MitraClip® arm where it was joined (Fig. 2).

### No vegetation was found on valves other than the mitral valve

**Fig. 3:**
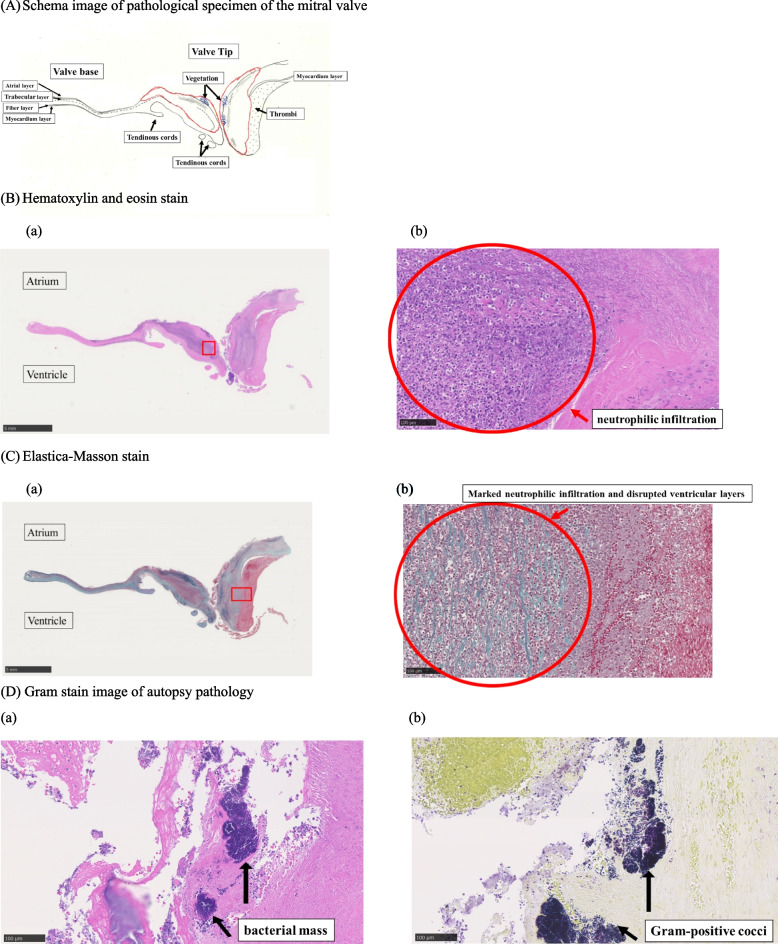
Histological findings of autopsy pathology. **A** Schema image of the pathological specimen of the mitral valve. **B** Hematoxylin–eosin (H-E) staining. **a** Overall view of the mitral valve with H-E staining. **b** Enlarged image of H-E staining showing neutrophilic infiltration, suggesting infection. **C** Elastica-Masson staining. **a** Overall view of the mitral valve with Elastica-Masson staining. **b** Magnified image of Elastica-Masson staining. The fibrous layer shows a high degree of neutrophilic infiltration, and the area including the ventricular layer is totally disrupted. Thrombi are adherent as a secondary change due to this effect. **D** Gram stain images of autopsy pathology. **a** An intensely magnified image of H-E staining showing a large amount of bacterial mass in the darkly stained area. **b** Gram staining of the same specimen showing Gram-positive cocci

The autopsy pathology showed severe destruction of the mitral valve. Hematoxylin and eosin (H-E) staining showed marked infiltration of neutrophils in the mitral valve tissue at the site of contact with the verruca and a large amount of bacterial mass in the darkly stained area of the valve tissue. Gram staining of the pathology specimen showed positive cocci, a finding consistent with MRSA cultured by various culture tests. Elastica-Masson staining (a staining method that clearly shows connective tissues such as elastic fibers and collagen fibers) also showed a high degree of neutrophilic infiltration in the atrial and trabecular layers, and the valve structure was destroyed. The fibrous layer showed a high degree of neutrophilic infiltration, and all layers, including the myocardial layer, were destroyed (Fig. 3).

## Discussion and Conclusions

Although there have been many reports of CDRIE of native valves, prosthetic valves, valves after TAVI, pacemakers, implantable cardioverter-defibrillators, and biventricular pacemakers, there have been only a few reports of IE after MitraClip® implantation [16–18]. Furthermore, to the best of the authors' knowledge, there has been no autopsy report of CDRIE after MitraClip® implantation. There has also been no report showing the extent of tissue damage caused by IE in histopathology.

Autopsy pathology findings also showed Gram-positive cocci. Clinical findings suggested that MRSA had entered the vessels percutaneously through the tunnel hemodialysis catheter or tunnel infection around the catheter, leading to bacteremia and IE.

Prior to MitraClip® implantation, the patient had severe mitral regurgitation and poor fluid management. Tricuspid valve regurgitation was also functionally severe. In view of his general condition, a decision was made to perform MitraClip® implantation instead of surgical intervention. After MitraClip® implantation, the MR became mild and cardiac output increased. The problem of difficulty in hemodialysis was resolved and fluid management was good. Tricuspid regurgitation also improved from severe to mild. The clinical course demonstrated that IE occurred only in the mitral valve, which may have been related to the recent implantation of the MitraClip® [19].

The MitraClip® was covered with neointima and the intrinsic mitral valve itself was strongly disrupted in the autopsy specimen. There was thick vegetation mainly on the middle scallop joined by the MitraClip®. The vegetation on the middle scallop had spread to the medial and lateral sides and also extended toward the left ventricle through the two unjoined and open native mitral valve entries. The synergistic effect of placing the Mitraclip® between the middle scallop of the anterior leaflet and the middle scallop of the posterior leaflet in a compromised host may have caused bacteria to adhere to the central area of the native mitral valve, creating an environment in which colonies could easily form and proliferate.

H-E staining of the autopsy pathology specimen showed marked neutrophilic infiltration, and Gram-positive cocci were detected by Gram staining despite antibiotic treatment. Elastica-Masson staining showed a high degree of neutrophilic infiltration in the atrial and trabecular layers and a high degree of neutrophilic infiltration in the fibrous layer toward the valve cusps, indicating that the valve structure was destroyed in all layers including the ventricular layer [20].

Our patient underwent the less invasive MitraClip® procedure for functional MR, which makes it difficult to maintain hemodynamics during dialysis. However, our patient had low cardiac function and was an easily infected host dialyzed with an indwelling dialysis catheter rather than an internal shunt, which predisposed him to CDRIE and IE. We hypothesized that the verruca was refractory to antibiotic therapy after the onset of IE and further increased in size. In addition, histopathology confirmed that there was significant tissue destruction due to IE.

In conclusion, even in cases in which highly invasive treatment such as mitral valvuloplasty or mitral valve replacement is not feasible, the indication for even minimally invasive treatment with MitraClip® should be carefully considered in compromised hosts such as our patient. In addition, we should consult doctors in an infectious disease department and consider performing the MitraClip® procedure after screening for MRSA or antibiotic-resistant bacteria and administering appropriate antibiotic prophylaxis.

## Data Availability

Please contact the corresponding author regarding data availability. The Ethics Committee will review the request to determine whether it is reasonable and whether the data can be shared.

## Abbreviations

CDRIE: Cardiac device-related infective endocarditis
MRSA: Methicillin-resistant *Staphylococcus aureus*
H-E: Hematoxylin and eosin
